# Expression profiling pre-diabetic mice to uncover drugs with clinical application to type 1 diabetes

**DOI:** 10.1038/cti.2015.17

**Published:** 2015-08-28

**Authors:** Dimeng Pang, Katharine M Irvine, Ahmed M Mehdi, Helen E Thomas, Mark Harris, Emma E Hamilton-Williams, Ranjeny Thomas

**Affiliations:** 1 Diamantina Institute, Translational Research Institute, University of Queensland, Woolloongabba, Queensland, Australia; 2 St Vincent's Institute, Fitzroy, Victoria, Australia; 3 Department of Paediatric Endocrinology, Mater Health Services, South Brisbane, Queensland, Australia

## Abstract

In the NOD mouse model of type 1 diabetes (T1D), genetically identical mice in the same environment develop diabetes at different rates. Similar heterogeneity in the rate of progression to T1D exists in humans, but the underlying mechanisms are unclear. Here, we aimed to discover peripheral blood (PB) genes in NOD mice predicting insulitis severity and rate of progression to diabetes. We then wished to use these genes to mine existing databases to identify drugs effective in diabetes. In a longitudinal study, we analyzed gene expression in PB samples from NOD.CD45.2 mice at 10 weeks of age, then scored pancreatic insulitis at 14 weeks or determined age of diabetes onset. In a multilinear regression model, *Tnf* and *Tgfb* mRNA expression in PB predicted insulitis score (*R*^2^=0.56, *P*=0.01). Expression of these genes did not predict age of diabetes onset. However, by expression-profiling PB genes in 10-week-old NOD.CD45.2 mice, we found a signature of upregulated genes that predicted delayed or no diabetes. Major associated pathways included chromatin organization, cellular protein location and regulation of nitrogen compounds and RNA. In a clinical cohort, three of these genes were differentially expressed between first-degree relatives, T1D patients and controls. Bioinformatic analysis of differentially expressed genes in NOD.CD45.2 PB identified drugs that are predicted to delay or prevent diabetes. Of these drugs, 11 overlapped with drugs predicted to induce a human ‘non-progressor' expression profile. These data demonstrate that disease heterogeneity in diabetes-prone mice can be exploited to mine novel clinical T1D biomarkers and drug targets.

In autoimmune type 1 diabetes (T1D), insulin-producing β cells of the pancreatic islets of Langerhans are destroyed or dysfunctional, resulting in hyperglycemia. There is no cure for T1D, which develops in children and young adults, and is associated with multiple complications and early mortality.^1^ The non-obese diabetic (NOD) mouse model of autoimmune diabetes shares many features with human T1D, including a number of regions of genetic overlap, multiple common autoantigens, shared MHC structure, as well as a marked influence of the environment on incidence.^2^ Nevertheless, translation of conclusions drawn from an inbred mouse strain in a controlled environment, to outbred humans has proven problematic. A common feature in NOD mice and human T1D is the considerable heterogeneity in the age of onset of diabetes.^3^ While there are many genetic and environmental contributors to heterogeneity in humans,^4^ in NOD mice heterogeneity in age of onset occurs among inbred, genetically-susceptible females even within the same breeding colony, where environmental differences are minimized. Inflammation of the islet (insulitis) begins predictably, soon after birth in all mice. However, overt diabetes development is variable, occurring from around 12 weeks of age in some mice, but much later or not at all in others.^5^

A major goal in diabetes research is to develop novel therapies, which target the mechanisms underlying disease.^6^ The observed heterogeneity in pre-clinical progression to T1D suggests a spread in the spectrum of disease immunopathogenesis.^3, 7^ This heterogeneity has seriously impacted the outcome of immunotherapy trials in T1D, as phase III trials of treatments given at diabetes onset have failed to meet their primary end points. *Post hoc* analysis suggests that heterogeneity in the population affects response and may be reducing power to achieve clinical end points.^8, 9^ In NOD mice, if mechanisms underlying the variability in age of diabetes onset under controlled environmental conditions were uncovered, then they could be exploited to identify potential novel therapies for clinical application to T1D.

Previous studies have investigated whether T-cell markers or magnetic resonance imaging could be used to predict the age of diabetes onset in NOD mice. The positive predictive value of the frequency of peripheral blood (PB) CD8 T cells specific for IGRP (islet-specific glucose-6-phosphate catalytic subunit-related protein) was around 85%, for age of onset. However, multiple eye bleeds were necessary to identify the rise in IGRP-specific cells.^10, 11^ A non-invasive method using magnetic resonance imaging of magnetic nanoparticles in 6- to 10-week NOD mice predicted the age of future diabetes onset.^12^ This methodology has been translated to human clinical cohorts.^13^ Mice protected from diabetes were found to have larger numbers of myeloid cells infiltrating the pancreatic islets, and transcriptomic analysis suggested that their phagocytic function played an important regulatory role.^14^ Given that diabetes onset should be predictable in 10-week-old NOD mice, we aimed to discover PB genes predicting either insulitis severity or onset of diabetes in NOD mice, and to discover mechanisms underlying heterogeneity in rate of progression. We then wished to exploit the mechanistic insight gained from expression profiling to seek potential biomarkers in human and to mine existing databases to identify drugs with clinical application to T1D.

## RESULTS

### *Tnf* and *Tgfb* expression in PB of 10-week-old NOD mice is negatively correlated with insulitis but unrelated to diabetes onset

**Figure 1:**
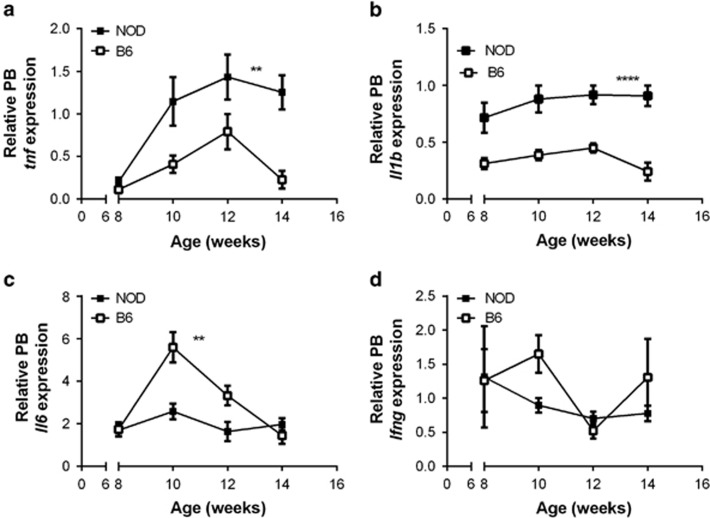
PB proinflammatory gene expression in NOD mice from 8 to 14 weeks of age. Expression of *Tnf* (**a**), *Il1b* (**b**), *Il6* (**c**) and *Ifng* (**d**) was determined by RT-PCR in PB sampled serially from female NOD.CD45.2 (*n*=17) and female C57BL/6 mice (*n*=5) between 8 and 14 weeks of age. Data are normalized to the housekeeping gene, HPRT and an internal plate control. Data are shown as mean and s.e.m. **P<0.01, ****P<0.0001 (two-way ANOVA in which the NOD.CD45.2 and C57BL/6 groups were compared over the time course).

NF-κB family members and their target proinflammatory cytokines have been shown to be overexpressed in the pre-clinical stage in NOD mice.^15, 16, 17^ We previously found time-dependent changes in *Il1b*, *Tnf, Ifng* and *Il6* mRNA expression in islets of NOD mice between 4 and 15 weeks of age.^18^ We first compared expression of these cytokines in PB of 8- to 14-week-old NOD.CD45.2 and C57BL/6 mice. We studied female NOD.CD45.2 mice from our breeding colony, which have a high proportion of mice with early onset of diabetes (Supplementary Figure 1A). RNA was successfully extracted from 50 to 100 μl of PB. We found that PB *Tnf* and *Il1b* expression was significantly increased and *Il6* expression significantly decreased in NOD.CD45.2 relative to age-matched C57BL/6 mice (Figures 1a and d). Given that diabetes onset was predictable by magnetic resonance imaging in 10-week-old NOD mice, we next looked for an association between insulitis or diabetes onset and NF-κB subunit or inflammatory gene expression in PB of 10-week-old NOD.CD45.2 mice.

After analysis of gene expression in PB at 10 weeks, we aged the mice to 14 weeks then scored the islets for infiltrate. Insulitis scores at 14 weeks varied in individual NOD.CD45.2 mice (Supplementary Figure 1B). After univariate linear regression of the relationship with insulitis at 14 weeks, features with *P*⩽0.065 were included in a multilinear regression model. A multiple linear regression model demonstrated a significant relationship between mean insulitis score and PB *Tnf* and *Tgfb* expression at 10 weeks (Table 1). To determine the relationship between gene expression and diabetes onset, we repeated the study with a second cohort of NOD.CD45.2 mice, quantifying PB gene expression at 10 weeks in the same way, then aging the mice to diabetes onset. Diabetes onset began around 100 days of age and 20% of the mice remained diabetes free by 400 days (Supplementary Figure 1A). A similar pattern of gene expression was observed. In univariate linear regression analysis, no PB gene was significantly associated with age of diabetes onset (data not shown).

### A distinct gene signature predicts diabetes onset in NOD mice

**Figure 2:**
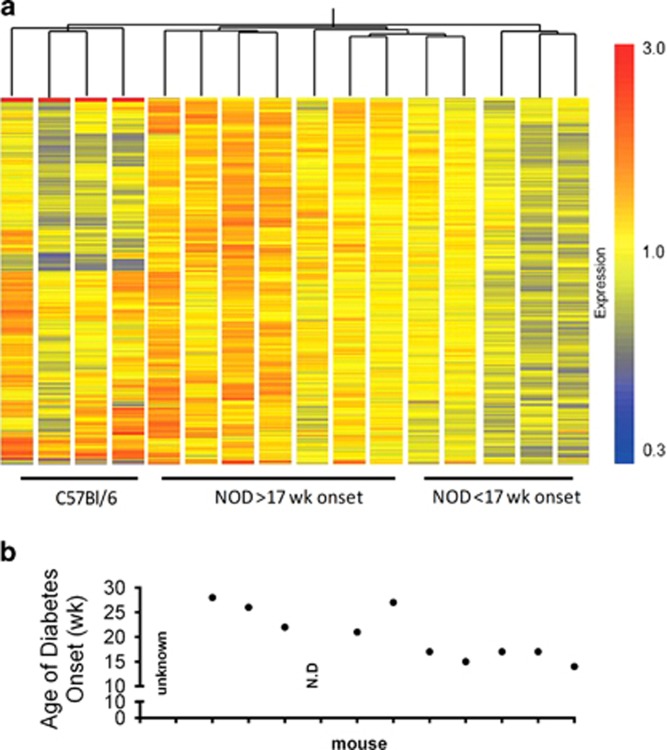
PB gene expression profiling at 10 weeks discriminates subsequent diabetes onset in NOD mice. (**a**) Illumina whole genome profiling of RNA extracted from PB of 10-week-old NOD.CD45.2 (*n*=12) and C57BL/6 mice (*n*=4). The genes (*n*=365) that were differentially expressed between mice developing diabetes before or after 17 weeks of age (*P*<0.05, *t*-test) and samples were clustered by distance correlation. (**b**) Age of diabetes onset for each mouse in this study, ‘Unknown' mouse died of unknown causes at 18 weeks, but was not diabetic. N.D., not diabetic at 34 weeks when experiment terminated.

To discover novel PB genes associated with diabetes onset using a hypothesis-free approach, we profiled global gene expression in PB of 12 female NOD.CD45.2 and 4 C57BL/6 mice at 10 weeks of age, then determined the age of diabetes onset in each NOD.CD45.2 mouse. Since approximately 50% of this cohort of NOD.CD45.2 mice developed T1D by 17 weeks of age, we first compared PB genes expressed by NOD.CD45.2 mice with diabetes onset at or before 17 weeks and those with diabetes onset after 17 weeks of age. There were 365 genes upregulated (*P*<0.05, Student's *t*-test) in mice with late-onset/no diabetes relative to early-onset diabetes, of which 151 were differentially expressed above a threshold fold change of 1.25 (Supplementary Table 1). The maximum fold change comparing groups was 1.8299. As the study was exploratory and analysis of whole blood may have blunted the magnitude of changes that might have been observed if purified blood cell populations had been analyzed, we explored genes differentially expressed above this relatively small fold difference. Figure 2 depicts differentially expressed genes in early- and late-onset groups, and the age of diabetes onset in each mouse. The expression of this geneset in PB from C57BL/6 mice is shown for comparison, and although there were evident differences between strains (in these and many other genes, not shown), the C57BL/6 expression pattern for these genes more closely resembled the late-onset NOD mice. We designated the group of 365 upregulated genes in late relative to early onset ‘G1', and assigned specific Gene Ontology (GO) terms. After correction for multiple hypothesis testing, the upregulated genes were significantly enriched in GO processes including metabolic processes, organelle organization, macromolecule methylation, RNA metabolism and processing, chromatin organization and modification, protein modification and T-cell proliferation (Table 2).

Gene expression was also analyzed against age of diabetes onset as a quantitative trait. The top nine genes correlating with age of onset are shown in Supplementary Table 2 (*P*<0.001). Some genes such as *Sirt7, Tbk1* and *Lamp2* were significantly overexpressed in the group developing diabetes after 17 weeks, but expression did not correlate with time of diabetes onset. However, other genes such *Vamp2, Chi3l3* and *Shc1* were both significantly overexpressed in mice developing diabetes late and expression correlated with time of diabetes onset (correlation coefficients 0.841, 0.681 and 0.718; *P*-values 0.004, 0.035 and 0.024, respectively). The gene most significantly correlated with age of diabetes onset was *Dag1* (*r*=0.976, *P*<1e−07).

### Individual PB genes predictive of diabetes onset

**Figure 3:**
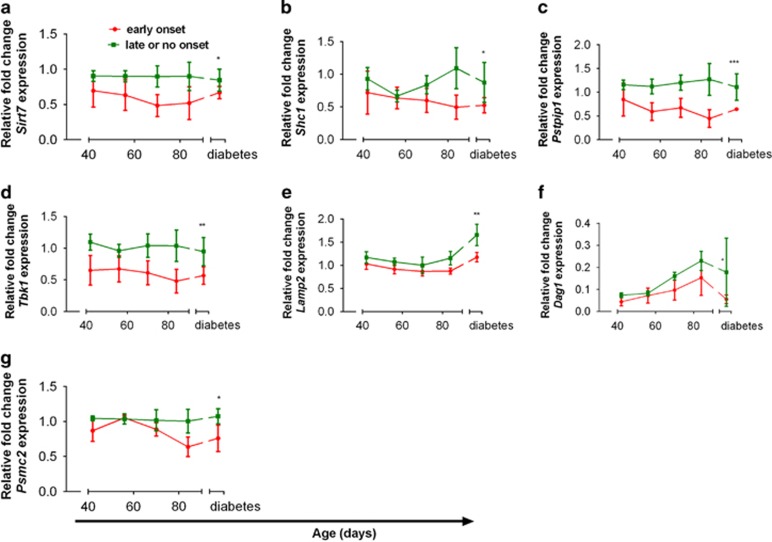
Validation of longitudinal PB gene expression by qPCR in NOD.CD45.2 mice. The relative fold expression of a selection of the top differentiated genes from the microarray was measured by qPCR in PB obtained at 2-week intervals from NOD.CD45.2 mice between 6 and 12 weeks of age, then at diabetes onset. (**a**) *Sirt7*, (**b**) *Shc1*, (**c**) *Pstpip1*, (**d**) *Tbk1*, (**e**) *Lamp2*, (**f**) *Dag1* and (**g**) *Psmc2*. Mice were grouped based on time of diabetes onset, either early (⩽17 weeks, red lines) or late or no diabetes (*n*=5–6 per group, green lines). Data were analyzed by two-way ANOVA. Data represent mean±s.e.m. *P<0.05, **P<0.01, ***P<0.001.

On the basis of a combination of fold change and potential biological role in T1D pathogenesis, 9 genes—*Chi3l3*, *Lamp2*, *Vamp2*, *Sirt7*, *Tbk1*, *Pstpip1*, *Psmc2*, *Shc1* and *Dag1*—were selected for validation in PB in an independent cohort of 13 NOD.CD45.2 mice between the ages of 6 and 12 weeks (genes highlighted in Supplementary Table 1). In this cohort, five mice developed diabetes at or before 17 weeks, six mice developed diabetes after 17 weeks and 2 did not develop diabetes. Two genes, *Vamp2* and *Chi3l3*, were not significantly differentially expressed in mice according to diabetes onset before or after 17 weeks of age (data not shown). However, *Sirt7* (*P*=0.0115), *Pstpip1* (*P*=0.0007), *Tbk1* (*P*=0.0015), *Lamp2* (*P*=0.0074), *Psmc2* (*P*=0.0269), *Shc1* (*P*=0.0475) and *Dag1* (*P*=0.0410) were significantly lower in the group with onset before 17 weeks than the late-onset group, replicating the microarray data (Figure 3). In some cases, gene expression differed significantly between groups from early in the disease course, for example, *Tbk1* from 6 weeks of age, and in other cases expression diverged later, for example, *Lamp2* at 12 weeks and diabetes onset (Figure 3).

### Cell-type expression of differentially-expressed genes in PB of NOD mice

**Figure 4:**
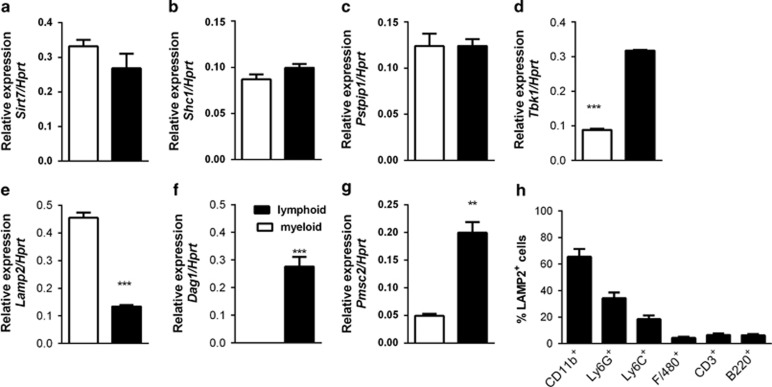
Cell population-specific expression of the genes discovered by microarray. Blood from 9- to 10-week-old NOD.CD45.2 mice (*n*=7) was pooled and sorted into CD11b+ myeloid cells and CD3^+^ or B220^+^ lymphoid cells. Expression of selected genes was quantified by qPCR (**a**) *sirt7*, (**b**) *shc1*, (**c**) *pstpip1*, (**d**) *tbk1*, (**e**) *lamp2*, (**f**) *psmc2* and (**g**) *dag1*. Relative expression of *Hprt* was used to normalize the data. **P<0.01, ***P<0.001 (*t*-test). (**h**) Fresh PB from individual 7- to 10-week-old NOD.CD45.2 female mice (*n*=10) was stained with a panel of surface markers and intracellular LAMP2, and then analyzed by flow cytometry. After gating on the LAMP2^+^ cells, percentages expressing each marker were calculated. ***P<0.001 (one-way ANOVA with Bonferroni's Multiple Comparison Test).

To determine whether the genes differentially expressed in relation to diabetes onset were predominantly expressed by myeloid or lymphoid cells, we pooled PB from seven NOD.CD45.2 mice aged 9–10 weeks and sorted myeloid and lymphoid cells by surface expression of CD11b and CD3/B220, respectively, then analyzed gene expression by qPCR. *Sirt7*, *Shc1* and *Pstpip1* were equally expressed by myeloid and lymphoid populations, *Tbk1, Psmc2* and *Dag1* were preferentially expressed by lymphocytes and *Lamp2* was preferentially expressed by myeloid cells (Figure 4). By intracellular flow-cytometry analysis, we confirmed that LAMP2 was expressed by CD11b^+^ Ly6G^+^ PB granulocytes (Figure 4h). It is likely that the difference in expression of the lymphocyte-enriched genes *Psmc2, Dag1* and *Tbk1* in mice with early or late diabetes onset was under-estimated by the analysis in whole blood, in which lymphocytes are under-represented.

### Expression of Lamp2, Tbk1 and Dag1 by PB mononuclear cells of children at risk of developing T1D

**Figure 5:**
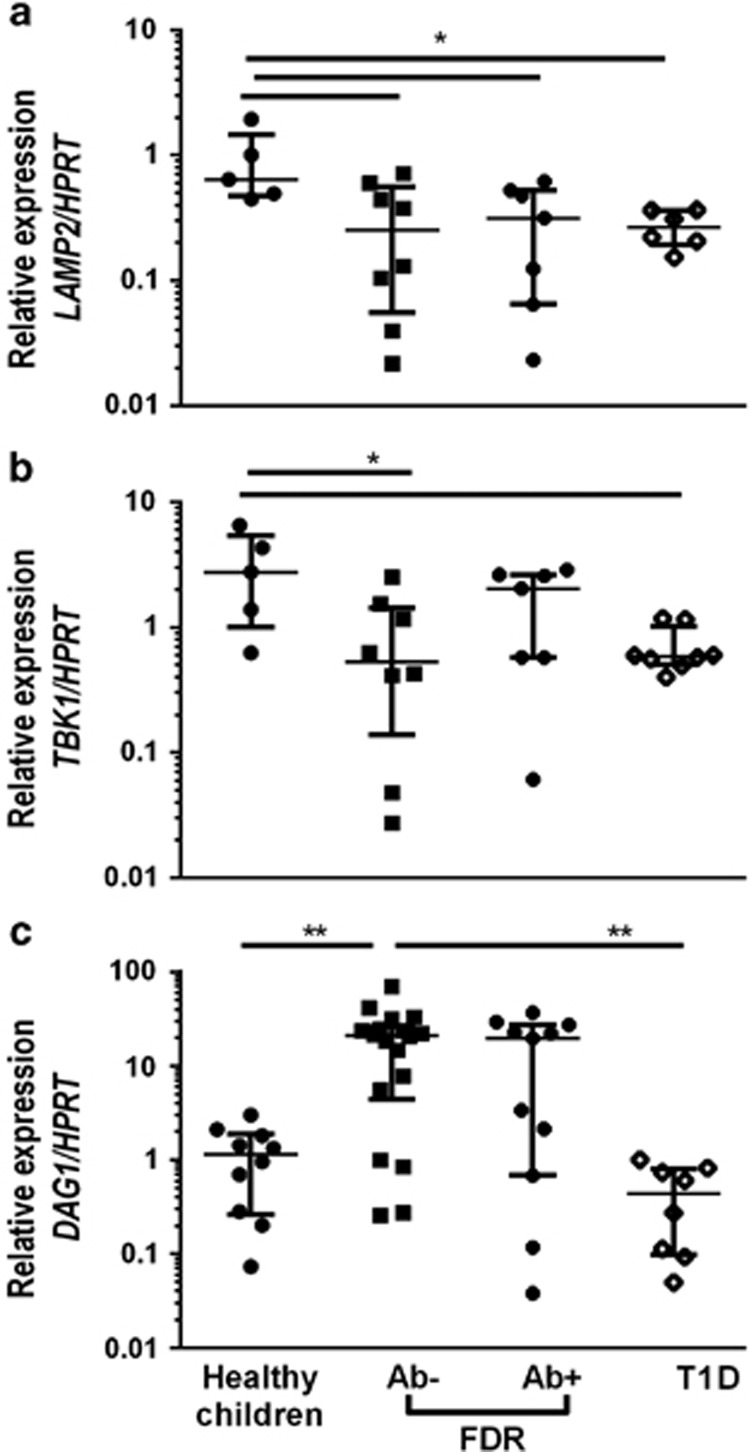
LAMP2, TBK1 and DAG1 are differentially expressed in children with or at risk of T1D and healthy controls. PBMC from children with recent-onset T1D (*n*=8), FDRs of T1D patients with (*n*=12) or without (*n*=17) autoantibodies, and age-matched healthy controls (*n*=12) was analyzed by qPCR to determine expression of LAMP2 (**a**), TBK1 (**b**) and DAG1 (**c**) relative to HPRT and an internal calibrator. The median and IQR are shown. *P<0.05, **P<0.01 (Kruskal–Wallis test with Dunn's multiple comparisons).

We then quantified mRNA expression for *LAMP2, TBK1* and *DAG1* in PB mononuclear cells (PBMCs) of children with recent-onset T1D (*n*=8), islet AB positive (*n*=11) and negative (*n*=18) first-degree relatives (FDRs) and unrelated healthy control children (*n*=12). *LAMP2* and *TBK1* showed a similar trend to each other, in that expression was higher in healthy controls than in FDR or recent-onset T1D patients. In contrast, *DAG1* was expressed at significantly higher levels by AB-negative FDR than either recent-onset T1D patients or healthy controls (Figure 5). These data indicate that the genes identified in NOD are also differentially expressed in children with different levels of risk of T1D.

### Development of a connectivity map to discover new drugs for T1D

Connectivity maps have been produced previously, to establish functional connections between genes, drugs and disease using a reference data set of gene-expression profiles from five human cancer cell lines treated with FDA (Food and Drug Administration)-approved drugs available at ArrayExpress database.^19, 20, 21^ To look for novel drugs that could delay or prevent the onset of diabetes in NOD mice, we interrogated the gCMAP package (with default parameters) to determine which small molecules promote upregulation of the genes in G1 within the reference data set.^22^ Drugs that induce expression of G1 genes with false discovery rate <0.1 are shown in Table 3, with their concentration when delivered to cell lines *in vitro*. Notably, several of these have been validated to prevent or arrest diabetes in NOD mice (imatinib, histone deacetylase (HDAC) inhibitors) or to improve c-peptide levels in children with recent-onset T1D and delay the onset of T1D in glucose-intolerant high-risk subjects (cyclosporine). Others are novel potential therapeutics in subjects at risk of T1D. To further test their relevance to human T1D, we re-interrogated the gCMAP package with each of the top 27 genes which Jin *et al.*^23^ found to classify risk of progression to T1D among islet antibody-positive subjects from the DAISY longitudinal cohort (Supplementary Table 3). We found an overlap of 11 drugs from mouse and human expression data (Table 4), which would be predicted to increase the expression of predictive genes expressed at low levels among diabetes progressors relative to non-progressors. These results further suggest that these drugs have potential to impact progression to T1D in at-risk subjects.

## DISCUSSION

This study identified a signature of upregulated gene expression in PB of pre-diabetic NOD mice, which predicts the likelihood of late or no diabetes onset. Expression of *Tnf* and *Tgfb1* in PB at 10 weeks of age was significantly negatively correlated with mean insulitis score. However, inflammatory gene expression in PB was not associated with diabetes onset. Diabetes onset reflects a functional end point that may not necessarily be correlated with a histological end point, that is, insulitis. The data suggest that different mechanisms influence islet inflammation and the speed of diabetes development in NOD mice. The negative associations between *Tnf, Tgfb1* and insulitis, suggest that regulatory processes induced in the context of systemic inflammation drive protective mechanisms in the islet. Indeed, the negative association between PB *Tgfb1* expression at 10-week and 14-week insulitis scores is consistent with previous reports demonstrating that islet protection by Foxp3^+^ regulatory T cells (Treg) is controlled by TGF-β.^24^ Consistent with this, a high frequency of intra-islet Treg has been demonstrated for up to 3 weeks after diabetes onset in NOD mice.^25^ Also consistent with our data, treatment of NOD mice with TNF during the insulitic phase suppressed diabetes development.^26^

NOD mice developing diabetes later or escaping diabetes development had an upregulated gene expression profile compared with mice developing diabetes before 17 weeks. The PB microarray of NOD mice at 10 weeks of age confirms a previous report suggesting that diabetes onset is already programmed at this age,^14^ and that pancreatic macrophages may protect against diabetes development. The late-onset or protective expression signature was enriched in pathways involving protein localization, cytoplasmic membrane-bounded vesicles, the proteasome complex, and protein catabolic processes. Of the genes validated by qPCR in a second cohort of NOD mice, *Pmsc2, Lamp2* and *Tbk1* are expressed in antigen-presenting cells and involved in antigen presentation and NF-κB signaling, reflecting similar processes to the previously identified macrophage pathways associated with protection. In contrast, upregulation of *Dag1*, *Sirt7* and *Shc1* might affect metabolic processes that modulate beta-cell growth, response to stress or insulin signaling.^27, 28, 29, 30, 31, 32, 33, 34, 35, 36^ Our data support the concept that molecular changes in immune and stromal cells influence the age of diabetes onset in mice with identical genetic background. In this regard, it is noteworthy that chromatin remodeling with the HDACi, trichostatin A, reduced the incidence of diabetes when delivered in the pre-diabetic period.^37^

To explore how the age of diabetes onset in NOD mice could be delayed, we used the connectivity map to determine drugs with the capacity to upregulate G1 genes. We identified 19 novel drugs with such effects. The capacity of several of these drugs or drug classes to delay or prevent the onset of diabetes or to reverse established diabetes has already been validated in NOD mice, including HDACi (trichostatin A and valproate) and the tyrosine-kinase inhibitor imatinib.^24, 38^ Indeed, imatinib and other tyrosine kinase inhibitors, including sunitinib led to reversal of hyperglycemia in NOD mice, potentially through effects on PDGFR signaling, and imatinib treatment for 10 weeks led to sustained remission for at least 35 weeks in 50% of treated mice.^38, 39^ Furthermore, cyclosporine A has been shown to increase the rate of remission and reduce the rate of C-peptide decline in children with recent-onset T1D, and to delay onset of T1D in glucose-intolerant pre-diabetic children.^40, 41^ Along similar lines to the HDACi, I-BAT151, an inhibitor of bromodomain-containing transcriptional regulators, which promotes chromatin remodeling, prevented diabetes development when delivered from 3 to 5, or 12 to 14 weeks of age, and reversed recent-onset diabetes in NOD mice.^42^ Of interest, further connectivity map screening of individual genes recently associated with progression to T1D in a longitudinal study of children, identified another 41 drugs, of which 11 overlapped with the drugs uncovered by the NOD mouse screen. Together, these studies identify novel drugs that could be used in proof-of-concept studies in NOD mouse models. Furthermore, repurposing of suitable compounds already approved for other indications is feasible for clinical trials in patients with or at risk of T1D. The current studies demonstrate a novel experimental path to uncover potential therapeutic candidates.

As a result of studying a limited selection of genes differentially expressed in PB of NOD mice, we identified significantly different levels of expression of *LAMP2, TBK1* and *DAG1* in children with recent-onset T1D relative to FDR and healthy controls. While longitudinal studies would be needed to determine the predictive value of these genes for diabetes onset in humans, our data demonstrate that studies exploring the heterogeneity of diabetes onset in NOD mice can also identify novel translatable PB biomarkers that may stratify risk in humans. A recent publication also supports the contention that genes overexpressed in NOD mice may be relevant prognostic biomarkers in children at risk of T1D.^43^ Thus, studies of disease heterogeneity in NOD mice may prove useful in identifying novel clinical T1D biomarkers and potential drug targets.

## METHODS

### Mice

NOD.Lt and C57BL/6 mice were obtained from the Animal Research Centre (Perth, Australia) and NOD.CD45.2 mice^44^ were bred at the University of Queensland. Experimental mice were housed under specific pathogen-free conditions at the biological research facility, Princess Alexandra Hospital (Brisbane, Australia). All experiments were approved by the University of Queensland animal ethics committee.

### Assessment of diabetes and insulitis

Urinary glucose was monitored twice weekly from 12 weeks of age using Diastix Reagent Strips (Bayer, Pymble, NSW, Australia). Glycosuric mice were tested for blood glucose using Accu-Chek Go System meters (Roche, Dee Why, NSW, Australia). Mice were classified as diabetic and killed following two consecutive blood glucose readings >15 mm. For analysis of insulitis, pancreata were collected, fixed in formalin and embedded for routine histology. Insulitis was graded in hematoxylin and eosin (H&E) stained sections on a scale of 0–4 as described, in at least 25 islets.^45^ Data are expressed as the proportion of islets of the total with each score.

### Quantitative real-time PCR

Serial samples of PB were collected by eye bleed, submandibular bleed or cardiac bleed into RNAlater solution (Ambion, Scoresby, VIC, Australia) then RNA was extracted (Ribo-Pure Blood kit, Ambion). Complementary DNA was synthesized from tissue RNA (Bioline reverse transcriptase). Real-time PCR used the SensiMix SYBR Hi-ROX Kit or SensiMix II Probe Kit (Bioline, Alexandria, NSW, Australia). Commercial TaqMan probe assays (Applied Biosystems, Scoresby, VIC, Australia) used in this study were mouse *Hprt* (assay ID: Mm00446968_m1), mouse *Tnf* (assay ID: Mm00443258_m1), mouse *Pmsc2* (assay ID: Mm00803207_m1), mouse *Sirt7* (assay ID: Mm01248607_m1), mouse *Shc1* (assay ID: Mm00468942_g1), mouse *Vamp2* (assay ID: Mm01325243_m1), mouse *Pstpip1* (assay ID: Mm00803222_m1), mouse *Tbk1* (assay ID: Mm00451150_m1), mouse *Lamp2* (assay ID: Mm00495267_m1), mouse *Chi313* (assay ID: Mm00657889_mH), mouse *Relb* (assay ID: Mm00485664_m1), mouse *Tgfb* (assay ID: Mm00436955_m1) and mouse *Dag1* (Mm00802400_m1). We used an AB 7900 (Applied Biosystems) thermal cycler with the following amplification conditions: 45 cycles of 95 °C for 15 s and 60 °C for 1 min. Data were analyzed in triplicates using the delta-delta Ct method, with *hprt* as the housekeeping gene and the same calibrator sample was included in all plates.

### RNA extraction and microarray

Blood was collected retro-orbitally at 10 weeks of age from female NOD.CD45.2 and C57Bl/6 mice and stored in RNA*later* solution (Ambion) at −80 °C. RNA was extracted using Ribo-Pure Blood kit (Ambion), the RNA integrity and concentration were measured using 2100 Bioanalyzer (Agilent, Santa Clara, CA, USA). RNA was depleted of alpha and beta globin mRNA using GLOBINclear (Ambion) before amplification with TotalPrep RNA Amplification Kit (Illumina). Resulting cRNA was hybridized to MouseRef-8 v2.0 Expression BeadChip (Illumina).

### Patient information and PBMC extraction

Eight children with T1D aged 9.8±6.3 years (mean±s.d.) diagnosed within the last 3 months were recruited at the Mater Children's Hospital during 2012. The diagnosis of T1D was based on the typical clinical symptoms and auto-antibody positivity (GAD, IA-2, insulin, tested by radioimmunoassay). Ten healthy age and sex-matched children without a family history of autoimmune disease aged 7.7±4.8 years were recruited from operation list patients awaiting non-urgent surgery. FDRs of T1D patients included 11 autoantibody (AB)-positive and 16 AB-negative individuals, who were 7.8±2.9 and 9.4±3.8 years old, respectively. Informed consent was obtained from all participants, and the studies were approved by the Mater Health Services and University of Queensland Research Ethics Committees. PBMCs were extracted from whole blood and RNA was immediately purified using RNeasy Mini Kit, Qiagen (https://www.qiagen.com/us).

### Microarray analysis and data mining

Data were analyzed using Genespring GX (Agilent) and BRB Array Tools. Raw gene expression data were imported into Genespring and normalized to 50th percentile (per array) and each gene was normalized to its median expression across all arrays. Data were filtered to exclude probes that did not achieve an Illumina Detection Score of 1 in at least one array. Genes that were differentially expressed between mice with onset ⩽17 weeks and mice with onset ⩾18 weeks were identified by *t*-test (⩾1.25-fold, *P*<0.05, *n*=365 genes). Genes correlating with week of diabetes onset were identified using the Quantitative Trait tool in BRB Array Tools (Spearman correlation, *P*<0.001 for the univariate test, *n*=9). For gene ontology enrichment analysis, we filtered the group of genes upregulated in late relative to early onset (referred as G1) and the group that were not upregulated. We counted the number of genes in each group, distinguishing between genes assigned a specific GO term from those not assigned that GO term. Our null hypothesis was that GO terms of genes upregulated in late-onset diabetes would not differ from GO terms of genes not upregulated. The tests of hypergeometric distribution establish probability that the null hypothesis is correct. *P*-values corrected for multiple hypothesis testing (using Bonferroni correction) determined *E*-values. For connectivity mapping, we downloaded the reference data set of gene-expression profiles from five cancer human cell lines treated with FDA-approved drugs from the ArrayExpress database (ID: E-GEOD-5258). Using the gCMAP package in R, we investigated compounds producing upregulation/downregulation of G1 genes in the cell lines.

### Multiple linear regression model

We first performed univariate linear regression to identify gene expression associated with mean insulitis score. We then fitted a multiple linear regression model on features with *P*-value ⩽0.065 and |*R*|⩾0.50 identified in the univariate linear regression model. We reported the *P*-value for multiple linear regression model using F-statistics.

### Flow cytometry and cell sorting

Cardiac blood was pooled from seven female NOD.CD45.2 mice at 9–10 weeks of age. Blood was stained for CD11b PE, CD3 FITC and B220 FITC, all from Biolegend (San Diego, CA, USA), for 20 min at 4 °C. Cells were then washed with PBS and red blood cells lysed using ACK lysis buffer. Cells were sorted into CD3^+^B220^+^CD11b^−^ lymphoid and CD11b^+^CD3^−^B220^−^ myeloid populations using the MoFlo (Beckman Coulter, Indianapolis, IN, USA). For analysis of Lamp2 expression, fresh PB from individual 7- to 10-week-old NOD.CD45.2 female mice was stained with APC-labeled CD11b, Ly6G, Ly6C, F4/80, CD3 or B220 (all from Biolegend) and, after permeabilization, with PE-labeled anti-LAMP2/CD107b (Biolegend). Cells were analyzed by flow cytometry. The percentage of LAMP2^+^ cells within each gated population was calculated.

## Figures and Tables

**Table 1:** Multiple linear regression model of the relationship between PB cytokine gene expression at 10 weeks and mean insulitis score at 14 weeks in NOD mice

*Predictors*	*Estimate*	*s.e.*	*t-value*	*P (>|t|)*
Intercept	4.07	0.349	11.662	1.56 × 10^−7^
*Tnf*	−0.27	0.098	−2.760	1.86 × 10^−2^
*Tgfb*	−215.35	88.09	-2.501	2.94 × 10^−2^

Abbreviation: PB, peripheral blood. Regression coefficients, *t*-value, standard error (s.e.) and *P*-values are shown. After univariate linear regression, features with *P*⩽0.065 and |*R*|⩾0.50 were included in a multiple linear regression model. The Pearson regression coefficient for this model is *R*=0.75 (or multiple *R*^2^=0.56). The F-statistic *P*-value for the model is 0.01.

**Table 2:** Gene ontology (GO) categories enriched among genes that were upregulated in NOD mice with late-onset diabetes

*GO Term*	*Description*	*E-value*
GO:0006807	Nitrogen compound metabolic process	1.70E−03
GO:0016070	RNA metabolic process	6.33E−03
GO:1901360	Organic cyclic compound metabolic process	2.12E−02
GO:0045184	Establishment of protein localization	6.66E−02
GO:0071840	Cellular component organization or biogenesis	1.23E−01
GO:0006325	Chromatin organization	1.28E−01
GO:0035556	Intracellular signal transduction	1.39E−01
GO:0032259	Methylation	1.89E−01
GO:1902532	Negative regulation of intracellular signal transduction	2.18E−01
GO:0015031	Protein transport	2.12E−01
GO:0006464	Cellular protein modification process	2.19E−01
GO:0001510	RNA methylation	2.25E−01
GO:0042098	T-cell proliferation	2.27E−01

**Table 3:** The connectivity map of G1

*Set name*	*FDR*	*Hits (of 270)*	*Compound/drug*	*Dose (μM)*
237	5.83^−03^	59	N-(4-Aminobutyl)-5-chloro-2-naphthalenesulfonamide (W13)	1.0^−05^
198	6.29^−03^	77	Colchicine	1.0^−06^
221	2.93^−02^	14	Imatinib	1.0^−05^
189	3.01^−02^	66	Benserazide	1.0^−05^
220	3.63^−02^	64	Iloprost	1.0^−06^
193	3.63^−02^	11	Ciclosporin	1.0^−06^
199	3.63^−02^	16	Copper sulfate	1.0^−04^
229	4.81^−02^	14	Mesalazine	1.0^−04^
123	6.51^−02^	33	Trichostatin A	1.0^−07^
36	6.51^−02^	3	Blebbistatin	1.7^−05^
271	6.98^−02^	13	Thalidomide	1.0^−04^
177	6.98^−02^	22	3-[1-(p-Chlorobenzyl)-5-(isopropyl)-3-t-butylthioindol-2-yl]-2,2-dimethylpropanoic acid	1.0^−06^
17	6.98^−02^	42	2-(4-Morpholinyl)-8-phenyl-4H-1-benzopyran-4-one	1.0^−05^
150	7.51^−02^	43	Wortmannin	1.0^−08^
227	8.39^−02^	12	Mercaptopurine	1.0^−04^
140	9.42^−02^	10	Valproic acid	0.002
176	9.76^−02^	19	2-deoxy-D-glucose	0.01
183	9.76^−02^	14	Arachidonic acid	1.0^−05^
162	9.76^−02^	34	Rofecoxib	1.0^−05^

Abbreviation: FDR, false discovery rate. This table shows the drugs that can stimulate or reduce gene expression in G1 genes, 6 h post treatment of cell lines. The IDs of 270 genes (out of 365) were found in the reference data set.

**Table 4:** The connectivity map of 29 genes with predictive value for progression to T1D in the DAISY cohort, showing drugs in common with the connectivity map of G1

*Compound/drug*	*Pathway*	*Gene name*	*Normalized z-score*	*Log FC*	*Adj P*-*value*	*Dose*
2-(4-Morpholinyl)-8-phenyl-4H-1-benzopyran-4-one	PI3K inhibitor	ATP6V1F	11.11	−1.22	4.5^−04^	1.0^−05^
		PLD3	7.04	0.76	2.3^−02^	1.0^−05^
Benserazide	Aromatic-L-amino-acid decarboxylase inhibitor	ATP6V1F	11.16	−0.91	1.9^−03^	1.0^−05^
		PLD3	7.08	1.01	5.5^−03^	1.0^−05^
Colchicine	Microtubule assembly inhibitor	ATP6V1F	11.19	−0.74	6.9^−03^	1.0^−06^
		PLD3	7.12	1.24	2.4^−03^	1.0^−06^
Iloprost	Prostacyclin agonist	ATP6V1F	11.19	−0.75	6.6^−03^	1.0^−06^
		NFE2	4.73	0.87	7.4^−02^	1.0^−06^
		PLD3	7.01	0.60	7.1^−02^	1.0^−06^
Indomethacin^a^	COX inhibitor	ATP6V1F	11.21	−0.63	1.7^−02^	1.0^−04^
		PLD3	7.11	1.21	2.4^−03^	1.0^−04^
Mercaptopurine	Anti-metabolite	HP	5.84	2.28	3.7^−02^	1.0^−04^
		NFE2	5.27	2.49	9.2^−02^	1.0^−04^
Mesalazine	IBD anti-inflammatory	HP	5.84	2.32	3.5^−02^	1.0^−04^
		NFE2	5.27	2.71	5.2^−02^	1.0^−04^
N-(4-Aminobutyl)-5-chloro-2-naphthalenesulfonamide	Ca^2+^/calmodulin antagonist	EIF3A	8.10	1.01	1.9^−02^	1.0^−05^
		HP	6.14	2.16	2.2^−06^	1.0^−05^
		ITGB5	6.28	−0.59	4.5^−02^	1.0^−05^
		NFE2	5.38	2.77	4.1^−05^	1.0^−05^
		PLD3	7.32	−0.50	7.6^−02^	1.0^−05^
Trichostatin A	HDAC inhibitor	NFE2	5.11	2.26	1.1^−03^	1.0^−07^
Valproic acid	HDAC inhibitor	ATP6V1F	11.12	−1.19	4.4^−04^	1.0^−03^
		PLD3	7.03	0.70	3.3^−02^	1.0^−03^
Wortmannin	PI3K inhibitor	ATP6V1F	11.13	−1.10	4.4^−04^	1.0^−08^
		NFE2	5.10	2.21	1.2^−03^	1.0^−08^

Abbreviations: FC, fold change; HDAC, histone deacetylase; T1D, type 1 diabetes. This table shows the drugs in common with those in the connectivity map of G1 that can change gene expression 6 h post treatment of cell lines, in any of 29 genes identified as predictive for T1D progression, reported previously.^23^ Normalized expression levels, fold change (FC) and adjusted *P*-value are also shown. Pathways targeted by each drug or drug class are indicated.

a Same class of drug as rofecoxib.
