# Supplementary material for: Expression profiling pre-diabetic mice to uncover drugs with clinical application to type 1 diabetes
Source: Clin Transl Immunology. 2015 Aug 28;4(8):e41–. doi: 10.1038/cti.2015.17 (PMC4558439; doi:10.1038/cti.2015.17)
Supplement: Supplementary Information [file cti201517x1.docx]

# Supplementary data

**Suppl. Figure 1. Severity of insulitis in prediabetic NOD.CD45.2 mice at 14 weeks of age and incidence of diabetes in NOD.CD45.2 mice**

A: Survival curve of diabetes incidence among NOD.CD45.2 mice in our colony. B: The mice from the insulitis study were scored for insulitis at 14 weeks of age using the insulitis scoring system.


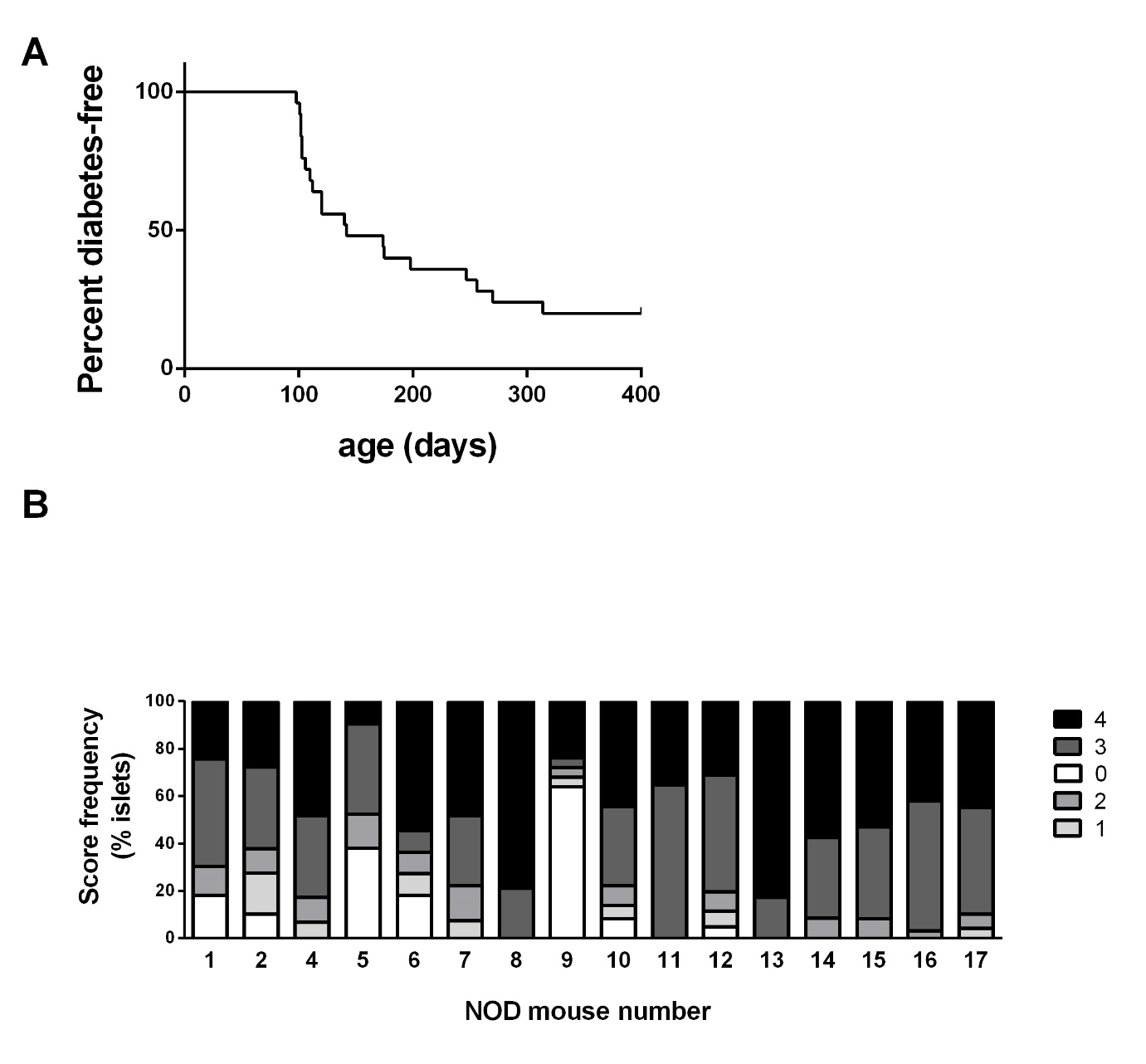


**Suppl. Table 1. Genes differentially expressed in PB of 10 week old NOD.CD45.2 mice developing diabetes before or after 17 weeks.** Genes identified by t test are included where the fold difference was >1.25. Genes highlighted in yellow were selected for further validation in another cohort of mice.

|  |  |  |  | Normalised expression | | |  |
| --- | --- | --- | --- | --- | --- | --- | --- |
| Illumina ID | Symbol | Genbank | Entrez GeneID | Average early onset | | Average late onset | Fold change |
| ILMN_226085 | Chi3l3 | NM_009892.1 | 12655 | 0.7331 | 1.3415 | | 1.8299 |
| ILMN_220109 | Pfc | XM_135820.3 |  | 0.9311 | 1.4694 | | 1.5782 |
| ILMN_214603 | Ddx3y | NM_012008.1 | 26900 | 0.8754 | 1.2976 | | 1.4824 |
| ILMN_209821 | Gdi1 | NM_010273.1 | 14567 | 0.8823 | 1.2959 | | 1.4688 |
| ILMN_214893 | Sepx1 | NM_013759.1 |  | 1.0157 | 1.4871 | | 1.4641 |
| ILMN_221263 | Adipor2 | NM_197985.2 |  | 0.9233 | 1.3258 | | 1.436 |
| ILMN_210446 | Lamp2 | NM_010685.2 |  | 0.8895 | 1.2747 | | 1.433 |
| ILMN_185188 | Vamp2 | NM_009497.2 |  | 0.8158 | 1.1557 | | 1.4167 |
| ILMN_190343 | Galt | NM_016658.1 |  | 0.9207 | 1.2792 | | 1.3893 |
| ILMN_210446 | Lamp2 | NM_010685.2 |  | 0.8736 | 1.2115 | | 1.3868 |
| ILMN_217242 | Slc38a2 | NM_175121.3 | 67760 | 0.8722 | 1.2085 | | 1.3856 |
| ILMN_201549 | Pira3 | NM_011090.1 | 18726 | 0.9903 | 1.369 | | 1.3823 |
| ILMN_217284 | Dok3 | NM_013739.1 |  | 0.796 | 1.0999 | | 1.3818 |
| ILMN_222389 | Gsdmdc1 | NM_026960.1 | 69146 | 0.907 | 1.2519 | | 1.3803 |
| ILMN_221041 | Nt5c | NM_015807.1 | 50773 | 0.9214 | 1.2701 | | 1.3784 |
| ILMN_261889 | Coq2 | NM_027978.1 | 71883 | 0.8315 | 1.1448 | | 1.3768 |
| ILMN_215550 | Sirt7 | NM_153056.1 | 209011 | 0.895 | 1.2282 | | 1.3723 |
| ILMN_211306 | Rga | NM_009057.1 | 19729 | 0.8449 | 1.1588 | | 1.3714 |
| ILMN_193247 | Slc11a1 |  |  | 0.877 | 1.2023 | | 1.3709 |
| ILMN_217174 | Mocs1 | NM_020042.1 | 56738 | 0.8323 | 1.1376 | | 1.3668 |
| ILMN_217477 | Rnf167 | NM_027445.1 | 70510 | 0.9045 | 1.2342 | | 1.3645 |
| ILMN_209726 | Adprtl2 | NM_009632.2 | 11546 | 0.7939 | 1.0798 | | 1.3602 |
| ILMN_217244 | Arl10b | NM_026823.1 |  | 0.8607 | 1.167 | | 1.356 |
| ILMN_210075 | Adssl1 | NM_007421.1 | 11565 | 0.8572 | 1.1618 | | 1.3554 |
| ILMN_259913 | Trappc1 | NM_001024206.1 | 245828 | 0.8089 | 1.0937 | | 1.352 |
| ILMN_218586 | Tbk1 | NM_019786.2 | 56480 | 0.886 | 1.1961 | | 1.35 |
| ILMN_193381 | Zfp263 | NM_148924.2 | 74120 | 0.8208 | 1.107 | | 1.3486 |
| ILMN_210491 | Pdlim4 | NM_019417.1 | 30794 | 0.9478 | 1.2777 | | 1.3481 |
| ILMN_213166 | 1110002B05Rik | NM_134054.1 |  | 0.8667 | 1.1679 | | 1.3476 |
| ILMN_236715 | Asb13 | NM_178283.3 | 142688 | 0.8742 | 1.1762 | | 1.3455 |
| ILMN_218880 | Anks3 | NM_028301.1 | 72615 | 0.9157 | 1.2318 | | 1.3451 |
| ILMN_222552 | Ell | NM_007924.1 |  | 0.9327 | 1.2541 | | 1.3446 |
| ILMN_220186 | Kctd2 | NM_183285.1 |  | 0.8885 | 1.1926 | | 1.3424 |
| ILMN_215958 | Pstpip1 | NM_011193.1 | 19200 | 0.8783 | 1.1782 | | 1.3415 |
| ILMN_216492 | Snx8 | NM_172277.1 | 231834 | 0.7963 | 1.0668 | | 1.3398 |
| ILMN_223340 | Acaa1 | NM_130864.2 |  | 0.9166 | 1.2279 | | 1.3396 |
| ILMN_218600 | 4833420G17Rik | NM_026127.3 | 67392 | 0.8891 | 1.1906 | | 1.339 |
| ILMN_211231 | Mgst2 | NM_174995.1 |  | 0.8913 | 1.1933 | | 1.3388 |
| ILMN_210645 | Bcas2 | NM_026602.1 |  | 0.8288 | 1.1093 | | 1.3384 |
| ILMN_213683 | Rbm5 | NM_148930.2 | 83486 | 0.846 | 1.132 | | 1.3381 |
| ILMN_208722 | AU067744 | NM_172529.1 |  | 0.8171 | 1.0929 | | 1.3376 |
| ILMN_221187 | Nit1 | NM_012049.1 | 27045 | 0.8786 | 1.1741 | | 1.3364 |
| ILMN_214107 | Dusp11 | NM_028099.2 | 72102 | 0.8694 | 1.1588 | | 1.3328 |
| ILMN_212010 | Arid2 | NM_175251.1 |  | 0.9033 | 1.202 | | 1.3306 |
| ILMN_218137 | Atp5g3 | NM_175015.1 | 228033 | 0.8435 | 1.1194 | | 1.3272 |
| ILMN_218893 | Plrg1 | NM_016784.2 | 53317 | 0.8709 | 1.1544 | | 1.3255 |
| ILMN_212794 | Fbxl12 | NM_013911.1 |  | 0.8772 | 1.1621 | | 1.3248 |
| ILMN_217462 | C730025P13Rik |  |  | 0.9195 | 1.2181 | | 1.3248 |
| ILMN_218161 | Ciz1 | NM_028412.1 | 68379 | 0.8432 | 1.1162 | | 1.3237 |
| ILMN_216498 | Il16 | NM_010551.1 |  | 0.8785 | 1.1622 | | 1.3231 |
| ILMN_213733 | 8430432M10Rik | NM_176831.2 |  | 0.8059 | 1.066 | | 1.3229 |
| ILMN_215956 | Prf1 | NM_011073.2 | 18646 | 0.9331 | 1.2343 | | 1.3227 |
| ILMN_186960 | Ss18 |  |  | 0.8684 | 1.1465 | | 1.3203 |
| ILMN_213982 | Centb1 | NM_153788.2 |  | 0.8345 | 1.1018 | | 1.3202 |
| ILMN_221050 | Mif4gd | NM_027162.3 | 69674 | 0.7806 | 1.03 | | 1.3194 |
| ILMN_213497 | Irgm | NM_008326.1 | 15944 | 0.8444 | 1.1117 | | 1.3166 |
| ILMN_208954 | Cope | NM_021538.1 | 59042 | 0.9147 | 1.2032 | | 1.3155 |
| ILMN_194092 | Ikbkap |  |  | 0.9855 | 1.2925 | | 1.3115 |
| ILMN_217454 | Itpr2 | NM_019923.2 |  | 0.7841 | 1.0283 | | 1.3114 |
| ILMN_211123 | Arpc1b | NM_023142.1 | 11867 | 0.9253 | 1.212 | | 1.3098 |
| ILMN_223488 | Psmc2 | NM_011188.1 | 19181 | 0.8774 | 1.1489 | | 1.3094 |
| ILMN_209821 | Gdi1 | NM_010273.1 | 14567 | 0.8074 | 1.0564 | | 1.3084 |
| ILMN_216302 | 0610039D01Rik | NM_026307.2 | 67675 | 0.8575 | 1.1213 | | 1.3076 |
| ILMN_213683 | Rbm5 | NM_148930.2 | 83486 | 0.8349 | 1.0909 | | 1.3066 |
| ILMN_190502 | 1500003D12Rik |  |  | 0.8724 | 1.1396 | | 1.3063 |
| ILMN_216855 | Ssr4 |  |  | 0.8789 | 1.1477 | | 1.3059 |
| ILMN_219285 | BC057552 | NM_172502.2 | 212123 | 0.9506 | 1.2404 | | 1.3048 |
| ILMN_212742 | Shc1 | NM_011368.3 | 20416 | 0.8872 | 1.1549 | | 1.3017 |
| ILMN_192190 | Zdhhc4 | NM_028379.1 | 72881 | 0.9288 | 1.2068 | | 1.2994 |
| ILMN_230229 | Adrbk1 | NM_130863.1 | 110355 | 0.8726 | 1.1335 | | 1.299 |
| ILMN_220480 | Slc25a28 | NM_145156.1 | 246696 | 0.8619 | 1.1189 | | 1.2982 |
| ILMN_215508 | 1110003E01Rik | NM_133697.1 |  | 0.8012 | 1.0381 | | 1.2957 |
| ILMN_210814 | Gorasp2 | NM_027352.2 | 70231 | 0.8597 | 1.1136 | | 1.2953 |
| ILMN_216512 | 2700060E02Rik | NM_026528.1 |  | 0.8521 | 1.1034 | | 1.2949 |
| ILMN_214901 | H2-Ke6 | NM_013543.1 | 14979 | 0.8719 | 1.1289 | | 1.2947 |
| ILMN_221666 | 0610040B21Rik | NM_025334.2 |  | 0.9125 | 1.181 | | 1.2943 |
| ILMN_212859 | Mfng | NM_008595.1 |  | 0.8228 | 1.0639 | | 1.293 |
| ILMN_212396 | Rai12 | NM_018740.1 | 54351 | 0.9697 | 1.2531 | | 1.2922 |
| ILMN_220751 | Rpp21 | NM_026308.1 | 67676 | 0.8974 | 1.159 | | 1.2915 |
| ILMN_216743 | Snx11 | NM_028965.2 | 74479 | 0.9117 | 1.1757 | | 1.2896 |
| ILMN_214618 | Etfa | NM_145615.2 | 110842 | 0.8663 | 1.1172 | | 1.2896 |
| ILMN_209902 | D8Ertd325e |  |  | 0.909 | 1.1719 | | 1.2892 |
| ILMN_209431 | Adprh | NM_007414.2 |  | 0.8527 | 1.099 | | 1.2889 |
| ILMN_220368 | Arl11 | NM_177337.3 | 219144 | 0.9903 | 1.2744 | | 1.2869 |
| ILMN_213953 | Atf4 | XM_139474.1 |  | 0.8951 | 1.151 | | 1.286 |
| ILMN_213203 | Klhdc2 | NM_027117.1 | 69554 | 0.885 | 1.1381 | | 1.286 |
| ILMN_215294 | Stxbp2 | NM_011503.2 |  | 0.8509 | 1.0934 | | 1.2849 |
| ILMN_211057 | 1810046J19Rik | NM_025559.1 |  | 0.8549 | 1.0981 | | 1.2845 |
| ILMN_211951 | Cdk5rap3 | NM_030248.1 | 80280 | 0.8773 | 1.1264 | | 1.2839 |
| ILMN_222663 | 1110012M11Rik | NM_028617.2 | 73711 | 0.9355 | 1.2011 | | 1.2839 |
| ILMN_222214 | Tmem66 | NM_026432.2 | 67887 | 0.8552 | 1.0979 | | 1.2838 |
| ILMN_211940 | Cxxc1 | NM_028868.1 | 74322 | 0.9113 | 1.1698 | | 1.2837 |
| ILMN_219426 | Tk2 | NM_021028.2 | 57813 | 0.9066 | 1.1627 | | 1.2826 |
| ILMN_222762 | Exosc8 | NM_027148.2 | 69639 | 0.8515 | 1.0921 | | 1.2825 |
| ILMN_218796 | Bri3 | NM_018772.1 |  | 0.8663 | 1.1096 | | 1.2809 |
| ILMN_194730 | Hsf1 |  |  | 0.8834 | 1.1311 | | 1.2803 |
| ILMN_191705 | Akr1b3 |  |  | 0.8789 | 1.1245 | | 1.2795 |
| ILMN_194153 | Preb |  |  | 0.9042 | 1.1565 | | 1.2789 |
| ILMN_213048 | Eif3s4 | NM_016876.2 |  | 0.8263 | 1.0567 | | 1.2788 |
| ILMN_210177 | 2410018C17Rik | NM_178390.2 | 74504 | 0.8701 | 1.1124 | | 1.2784 |
| ILMN_223118 | 0610041B22Rik | NM_025340.1 | 106025 | 0.9429 | 1.2051 | | 1.2781 |
| ILMN_220837 | Mlf2 | NM_145385.1 | 30853 | 0.8893 | 1.1366 | | 1.2781 |
| ILMN_222023 | Coro1b | NM_011778.1 | 23789 | 0.8788 | 1.1229 | | 1.2778 |
| ILMN_223596 | Mdh2 | NM_008617.2 | 17448 | 0.9135 | 1.1669 | | 1.2774 |
| ILMN_223238 | 3110001D03Rik | NM_025849.2 | 66928 | 0.8617 | 1.1001 | | 1.2767 |
| ILMN_211164 | D030070L09Rik | NM_172625.1 | 225280 | 0.9735 | 1.2427 | | 1.2766 |
| ILMN_223090 | AY078069 | NM_172142.1 | 243910 | 0.7856 | 1.0025 | | 1.2761 |
| ILMN_221941 | Hps3 | NM_080634.2 | 12807 | 0.9366 | 1.195 | | 1.2759 |
| ILMN_221506 | Bscl2 | NM_008144.3 | 14705 | 0.913 | 1.1648 | | 1.2758 |
| ILMN_217480 | Suclg1 | NM_019879.1 | 56451 | 0.8816 | 1.1248 | | 1.2757 |
| ILMN_208792 | D19Ertd721e | NM_146093.1 | 225896 | 0.8901 | 1.135 | | 1.2751 |
| ILMN_195929 | Zdhhc12 | NM_025428.1 |  | 0.8847 | 1.1271 | | 1.2739 |
| ILMN_209328 | Pex6 | NM_145488.1 | 224824 | 0.8696 | 1.1064 | | 1.2723 |
| ILMN_185541 | Tpcn1 | NM_145853.2 | 252972 | 0.8676 | 1.1038 | | 1.2723 |
| ILMN_208917 | BC065078 | NM_201351.1 | 225912 | 0.8429 | 1.0718 | | 1.2716 |
| ILMN_221714 | Lmo6 | NM_175097.2 | 54630 | 0.9394 | 1.1944 | | 1.2714 |
| ILMN_221079 | Gps2 |  |  | 0.8851 | 1.1251 | | 1.2712 |
| ILMN_238375 | Htf9c | NM_001081000.1 | 15547 | 0.8696 | 1.1052 | | 1.2709 |
| ILMN_220818 | 1700012G19Rik | NM_025954.2 |  | 0.8918 | 1.1323 | | 1.2697 |
| ILMN_217880 | Ndufs7 | NM_029272.1 | 75406 | 0.8285 | 1.0515 | | 1.2691 |
| ILMN_191561 | Ube2q | NM_027315.2 | 70093 | 0.8922 | 1.1318 | | 1.2686 |
| ILMN_220165 | 1200009C21Rik | NM_028659.1 | 73830 | 0.8651 | 1.0974 | | 1.2686 |
| ILMN_221019 | Rpl29 | NM_009082.2 | 19944 | 0.8181 | 1.0375 | | 1.2682 |
| ILMN_193220 | 5033425B17Rik |  |  | 0.8799 | 1.1152 | | 1.2674 |
| ILMN_218550 | Phtf2 | NM_172992.2 | 68770 | 0.9956 | 1.2603 | | 1.2658 |
| ILMN_213412 | Lass2 | NM_029789.1 | 76893 | 0.8956 | 1.1335 | | 1.2657 |
| ILMN_210065 | Mrpl52 | NM_026851.1 | 68836 | 0.8395 | 1.0621 | | 1.2652 |
| ILMN_209825 | Mrpl28 | NM_024227.2 | 68611 | 0.84 | 1.0628 | | 1.2652 |
| ILMN_222120 | Gadd45g | NM_011817.1 | 23882 | 0.8872 | 1.1209 | | 1.2633 |
| ILMN_211860 | Cyp4f13 | NM_130882.1 | 170716 | 0.9008 | 1.1366 | | 1.2618 |
| ILMN_187550 | Timm10 |  |  | 0.8635 | 1.0882 | | 1.2603 |
| ILMN_218866 | 2410104I19Rik | NM_133691.1 |  | 0.8946 | 1.1271 | | 1.2599 |
| ILMN_213690 | Drap1 | NM_024176.1 | 66556 | 0.8211 | 1.0343 | | 1.2595 |
| ILMN_212989 | Psmc4 | NM_011874.1 |  | 0.873 | 1.0995 | | 1.2594 |
| ILMN_216598 | Mrps24 | NM_026080.1 |  | 0.9111 | 1.1466 | | 1.2585 |
| ILMN_213412 | Lass2 | NM_029789.1 | 76893 | 0.906 | 1.1395 | | 1.2577 |
| ILMN_248674 | 2510012J08Rik | NM_027381.1 | 70312 | 0.9456 | 1.1892 | | 1.2576 |
| ILMN_219048 | Kpnb1 | NM_008379.2 |  | 0.9041 | 1.1354 | | 1.2558 |
| ILMN_242502 | LOC622404 | NM_001037913.1 | 622404 | 0.8957 | 1.1247 | | 1.2557 |
| ILMN_222286 | A230050P20Rik | NM_175687.1 | 319278 | 0.8708 | 1.093 | | 1.2552 |
| ILMN_209195 | 1110061O04Rik | NM_026849.1 | 97287 | 0.9183 | 1.1526 | | 1.2551 |
| ILMN_208949 | Fbxw5 | NM_013908.1 |  | 0.9243 | 1.16 | | 1.255 |
| ILMN_209438 | BC008155 | NM_145410.1 |  | 0.8958 | 1.1238 | | 1.2546 |
| ILMN_215679 | Lypla2 | NM_011942.1 | 26394 | 0.8605 | 1.0788 | | 1.2537 |
| ILMN_221446 | 3110009E18Rik | NM_028439.1 | 73103 | 0.8848 | 1.1093 | | 1.2536 |
| ILMN_215589 | Irf3 | NM_016849.2 |  | 0.8649 | 1.0838 | | 1.2531 |
| ILMN_211560 | Hdgfrp2 | NM_008233.1 | 15193 | 0.8902 | 1.1152 | | 1.2528 |
| ILMN_210478 | 2500002L14Rik | NM_025607.2 |  | 0.905 | 1.1334 | | 1.2524 |
| ILMN_218519 | Erp29 | NM_026129.2 | 67397 | 0.8527 | 1.0674 | | 1.2519 |
| ILMN_211903 | 6720425G15Rik | NM_198165.1 |  | 0.9787 | 1.2237 | | 1.2504 |
| ILMN_214265 | 5730466P16Rik | NM_173453.1 | 216821 | 0.8853 | 1.1068 | | 1.2502 |

**Suppl. Table 2. The first nine genes which significantly correlate with age of diabetes onset.**

The genes are significant at the nominal p<0.001 level of the univariate test. Genes highlighted in yellow were also differentially expressed between early and late diabetes.

|  | **Correlation coefficient** | **Parametric p-value** | **FDR** | **UniqueID** | **Accession** | **Symbol** |
| --- | --- | --- | --- | --- | --- | --- |
| 1 | 0.976 | < 1e-07 | < 1e-07 | 2030546 | [NM_010017](http://www.ncbi.nlm.nih.gov/entrez/query.fcgi?db=Nucleotide&term=NM_010017) | [Dag1](http://www.ncbi.nlm.nih.gov/entrez/query.fcgi?cmd=search&db=gene&term=Dag1) |
| 2 | 0.926 | 0.00013 | 0.147 | 1990204 | [NM_177620](http://www.ncbi.nlm.nih.gov/entrez/query.fcgi?db=Nucleotide&term=NM_177620) | [Rin3](http://www.ncbi.nlm.nih.gov/entrez/query.fcgi?cmd=search&db=gene&term=Rin3) |
| 3 | 0.926 | 0.00013 | 0.147 | 940438 | [NM_010686](http://www.ncbi.nlm.nih.gov/entrez/query.fcgi?db=Nucleotide&term=NM_010686) | [Laptm5](http://www.ncbi.nlm.nih.gov/entrez/query.fcgi?cmd=search&db=gene&term=Laptm5) |
| 4 | 0.926 | 0.00013 | 0.147 | 3870608 | [NM_181417](http://www.ncbi.nlm.nih.gov/entrez/query.fcgi?db=Nucleotide&term=NM_181417) | [Csrp2bp](http://www.ncbi.nlm.nih.gov/entrez/query.fcgi?cmd=search&db=gene&term=Csrp2bp) |
| 5 | 0.914 | 0.000467 | 0.351 | 870274 | [NM_139272](http://www.ncbi.nlm.nih.gov/entrez/query.fcgi?db=Nucleotide&term=NM_139272) | [Galnt2](http://www.ncbi.nlm.nih.gov/entrez/query.fcgi?cmd=search&db=gene&term=Galnt2) |
| 6 | 0.914 | 0.000467 | 0.351 | 5690411 | [NM_175251](http://www.ncbi.nlm.nih.gov/entrez/query.fcgi?db=Nucleotide&term=NM_175251) | [Arid2](http://www.ncbi.nlm.nih.gov/entrez/query.fcgi?cmd=search&db=gene&term=Arid2) |
| 7 | 0.902 | 0.00088 | 0.441 | 4390239 | [NM_028715](http://www.ncbi.nlm.nih.gov/entrez/query.fcgi?db=Nucleotide&term=NM_028715) | [Fcho1](http://www.ncbi.nlm.nih.gov/entrez/query.fcgi?cmd=search&db=gene&term=Fcho1) |
| 8 | 0.902 | 0.00088 | 0.441 | 510669 | [NM_130882](http://www.ncbi.nlm.nih.gov/entrez/query.fcgi?db=Nucleotide&term=NM_130882) | [Cyp4f13](http://www.ncbi.nlm.nih.gov/entrez/query.fcgi?cmd=search&db=gene&term=Cyp4f13) |
| 9 | 0.902 | 0.00088 | 0.441 | 1070053 | [NM_013844](http://www.ncbi.nlm.nih.gov/entrez/query.fcgi?db=Nucleotide&term=NM_013844) | [Zfp68](http://www.ncbi.nlm.nih.gov/entrez/query.fcgi?cmd=search&db=gene&term=Zfp68) |

**Suppl. Table 3. The connectivity map of 29 genes identified by Jin and colleagues.**

The Table shows the drugs that can change gene expression in 29 genes identified by Jin and colleagues, 6 hours post-treatment of cell lines. Normalized expression levels, fold change and adjusted p-value are also shown.

| Compound / drug | Set Name | Gene  name | Normalized  z-score | Log FC | Adj  p-value | Cell Line | Dose |
| --- | --- | --- | --- | --- | --- | --- | --- |
| 15-delta prostaglandin J2 | 7 | ATP6V1F | 11.11 | -1.24 | 4.5^-04^ | SKMEL5 | 1.0^-05^ |
|  | 7 | PLD3 | 7.06 | 0.89 | 9.8^-03^ | SKMEL5 | 1.0^-05^ |
| 17-allylamino-geldanamycin | 8 | ETS2 | 6.26 | 0.47 | 1.2^-02^ | MCF7 | 1.0^-06^ |
|  | 11 | ATP6V1F | 11.12 | -1.17 | 4.5^-04^ | SKMEL5 | 1.0^-06^ |
|  | 10 | ATP6V1F | 10.13 | 0.55 | 1.2^-02^ | PC3 | 1.0^-06^ |
|  | 8 | ATP6V1F | 11.11 | 0.98 | 3.2^-02^ | MCF7 | 1.0^-06^ |
|  | 8 | ITGB5 | 6.72 | 0.46 | 7.5^-02^ | MCF7 | 1.0^-06^ |
|  | 11 | PLD3 | 7.08 | 1.01 | 5.5^-03^ | SKMEL5 | 1.0^-06^ |
| 2-(4-Morpholinyl)-8-phenyl-4H-1-benzopyran-4-one | 18 | ATP6V1F | 11.11 | -1.22 | 4.5^-04^ | SKMEL5 | 1.0^-05^ |
|  | 17 | ATP6V1F | 10.12 | 0.51 | 1.2^-02^ | PC3 | 1.0^-05^ |
|  | 18 | PLD3 | 7.04 | 0.76 | 2.3^-02^ | SKMEL5 | 1.0^-05^ |
|  | 15 | PLD3 | 7.30 | 0.28 | 8.6^-02^ | MCF7 | 1.0^-05^ |
| 4,5-dianilinophthalimide | 180 | ATP6V1F | 10.12 | 0.91 | 5.5^-03^ | PC3 | 1.0^-05^ |
| 5-(4-fluorophenyl)-1-[4-(methylsulfonyl)phenyl]-3-(trifluoromethyl)-1H-pyrazole | 156 | ATP6V1F | 11.12 | -1.18 | 4.5^-04^ | SKMEL5 | 1.0^-05^ |
|  | 156 | ITGB5 | 5.98 | 0.59 | 9.6^-02^ | SKMEL5 | 1.0^-05^ |
|  | 156 | PLD3 | 7.09 | 1.09 | 4.3^-03^ | SKMEL5 | 1.0^-05^ |
| 6-chloro-3-phenyl[1,2,4]triazolo[4,3-b]pyridazine | 31 | ETS2 | 6.22 | 1.35 | 5.5^-03^ | MCF7 | 1.0^-05^ |
| alpha-estradiol | 34 | ATP6V1F | 10.12 | 0.88 | 6.6^-03^ | PC3 | 1.0^-08^ |
| arotinoid acid | 186 | ATP6V1F | 10.11 | 0.82 | 9.9^-03^ | PC3 | 1.0^-07^ |
| benserazide | 189 | ATP6V1F | 11.16 | -0.91 | 1.9^-03^ | SKMEL5 | 1.0^-05^ |
|  | 189 | PLD3 | 7.08 | 1.01 | 5.5^-03^ | SKMEL5 | 1.0^-05^ |
|  |  |  |  |  |  |  |  |
| butein | 191 | ATP6V1F | 10.10 | 0.72 | 2.0^-02^ | PC3 | 1.0^-05^ |
| butirosin | 277 | ATP6V1F | 10.11 | 0.79 | 1.2^-02^ | PC3 | 1.0^-05^ |
| carbamazepine | 40 | ETS2 | 6.23 | 1.12 | 5.5^-03^ | MCF7 | 1.0^-07^ |
| colchicine | 198 | ATP6V1F | 11.19 | -0.74 | 6.9^-03^ | SKMEL5 | 1.0^-06^ |
|  | 198 | PLD3 | 7.12 | 1.24 | 2.4^-03^ | SKMEL5 | 1.0^-06^ |
| Dimethyloxalyl-glycine | 278 | HP | 5.81 | 1.99 | 8.3^-05^ | PC3 | 1.0^-03^ |
|  | 278 | NFE2 | 4.98 | 1.89 | 3.8^-03^ | PC3 | 1.0^-03^ |
|  |  |  |  |  |  |  |  |
| docosahexaenoic acid ethyl ester | 56 | ETS2 | 6.21 | 1.83 | 5.5^-03^ | MCF7 | 1.0^-04^ |
|  | 57 | HP | 5.81 | 1.90 | 1.0^-04^ | PC3 | 1.0^-04^ |
|  | 57 | ITGB5 | 6.25 | -0.54 | 9.7^-02^ | PC3 | 1.0^-04^ |
|  | 57 | NFE2 | 5.05 | 2.75 | 3.1^-04^ | PC3 | 1.0^-04^ |
| doxycycline | 58 | LHPP | 6.60 | -1.08 | 9.1^-03^ | MCF7 | 1.0^-05^ |
| estradiol | 62 | HP | 5.80 | 1.84 | 1.0^-04^ | PC3 | 1.0^-08^ |
|  | 62 | NFE2 | 5.03 | 2.50 | 4.7^-04^ | PC3 | 1.0^-08^ |
|  | 62 | PLD3 | 7.53 | -0.89 | 7.6^-02^ | PC3 | 1.0^-08^ |
| fasudil | 216 | NFE2 | 5.46 | 2.05 | 6.2^-02^ | PC3 | 1.0^-05^ |
|  |  |  |  |  |  |  |  |
| fisetin | 279 | HP | 5.84 | 2.23 | 3.8^-05^ | PC3 | 5.0^-05^ |
|  | 279 | ITGB5 | 6.25 | -0.54 | 9.6^-02^ | PC3 | 5.0^-05^ |
|  | 279 | NFE2 | 5.07 | 2.89 | 2.8^-04^ | PC3 | 5.0^-05^ |
|  |  |  |  |  |  |  |  |
| fluphenazine | 66 | PLD3 | 7.13 | 1.31 | 2.4^-03^ | SKMEL5 | 1.0^-05^ |
| fulvestrant | 69 | ATP6V1F | 10.11 | 0.74 | 1.7^-02^ | PC3 | 1.0^-06^ |
|  | 69 | HP | 5.72 | 0.95 | 2.9^-02^ | PC3 | 1.0^-06^ |
| genistein | 74 | ATP6V1F | 10.12 | 0.93 | 5.0^-03^ | PC3 | 1.0^-05^ |
| HC toxin | 76 | ETS2 | 6.21 | 1.62 | 1.7^-02^ | MCF7 | 1.0^-07^ |
| iloprost | 220 | ATP6V1F | 11.19 | -0.75 | 6.6^-03^ | SKMEL5 | 1.0^-06^ |
|  | 220 | NFE2 | 4.73 | 0.87 | 7.4^-02^ | SKMEL5 | 1.0^-06^ |
|  | 220 | PLD3 | 7.01 | 0.60 | 7.1^-02^ | SKMEL5 | 1.0^-06^ |
| indometacin | 226 | ATP6V1F | 11.21 | -0.63 | 1.7^-02^ | SKMEL5 | 1.0^-04^ |
|  | 226 | PLD3 | 7.11 | 1.21 | 2.4^-03^ | SKMEL5 | 1.0^-04^ |
|  |  |  |  |  |  |  |  |
| ionomycin | 78 | ETS2 | 6.23 | 0.98 | 1.2^-02^ | MCF7 | 2.0^-06^ |
| mercaptopurine | 227 | HP | 5.84 | 2.28 | 3.7^-02^ | MCF7 | 1.0^-04^ |
|  | 227 | NFE2 | 5.27 | 2.49 | 9.2^-02^ | MCF7 | 1.0^-04^ |
| mesalazine | 229 | HP | 5.84 | 2.32 | 3.5^-02^ | MCF7 | 1.0^-04^ |
|  | 229 | NFE2 | 5.27 | 2.71 | 5.2^-02^ | MCF7 | 1.0^-04^ |
| Methyl [5-methylsulfonyl-1-(4-chlorobenzyl)-1H-2-indolyl]carboxylate | 233 | HP | 5.88 | 1.81 | 7.5^-04^ | MCF7 | 1.0^-05^ |
|  | 233 | ITGB5 | 6.61 | -0.92 | 7.5^-02^ | MCF7 | 1.0^-05^ |
|  | 233 | NFE2 | 5.33 | 2.40 | 3.5^-04^ | MCF7 | 1.0^-05^ |
| monensin | 80 | ETS2 | 6.21 | 1.82 | 5.4^-03^ | MCF7 | 1.0^-05^ |
|  | 84 | ATP6V1F | 11.18 | -0.79 | 5.0^-03^ | SKMEL5 | 1.0^-07^ |
|  | 84 | NFE2 | 4.77 | 1.14 | 1.5^-02^ | SKMEL5 | 1.0^-07^ |
|  | 84 | PLD3 | 7.07 | 0.96 | 7.1^-03^ | SKMEL5 | 1.0^-07^ |
|  |  |  |  |  |  |  |  |
| N-(4-Aminobutyl)-5-chloro-2-naphthalenesulfonamide | 237 | EIF3A | 8.10 | 1.01 | 1.9^-02^ | MCF7 | 1.0^-05^ |
|  | 237 | HP | 6.14 | 2.16 | 2.2^-06^ | MCF7 | 1.0^-05^ |
|  | 237 | ITGB5 | 6.28 | -0.59 | 4.5^-02^ | MCF7 | 1.0^-05^ |
|  | 237 | NFE2 | 5.38 | 2.77 | 4.1^-05^ | MCF7 | 1.0^-05^ |
|  | 237 | PLD3 | 7.32 | -0.50 | 7.6^-02^ | MCF7 | 1.0^-05^ |
| novobiocin | 246 | ATP6V1F | 11.13 | -1.13 | 4.4^-04^ | SKMEL5 | 1.0^-04^ |
|  | 246 | PLD3 | 7.02 | 0.67 | 4.1^-02^ | SKMEL5 | 1.0^-04^ |
|  |  |  |  |  |  |  |  |
| pirinixic acid | 256 | ATP6V1F | 11.13 | -1.10 | 4.4^-04^ | SKMEL5 | 1.0^-04^ |
|  | 256 | PLD3 | 7.05 | 0.83 | 1.4^-02^ | SKMEL5 | 1.0^-04^ |
| sodium phenylbutyrate | 169 | ATP6V1F | 11.13 | -1.09 | 4.4^-04^ | SKMEL5 | 2.0^-04^ |
|  | 169 | PLD3 | 7.02 | 0.67 | 4.1^-02^ | SKMEL5 | 2.0^-04^ |
|  |  |  |  |  |  |  |  |
| staurosporine | 263 | ATP6V1F | 11.13 | -1.10 | 4.4^-04^ | SKMEL5 | 1.0^-08^ |
|  | 263 | PLD3 | 7.04 | 0.78 | 2.0^-02^ | SKMEL5 | 1.0^-08^ |
| trichostatin A | 123 | NFE2 | 5.11 | 2.26 | 1.1^-03^ | ssMCF7 | 1.0^-07^ |
| troglitazone | 128 | ATP6V1F | 11.11 | -1.23 | 4.4^-04^ | SKMEL5 | 1.0^-05^ |
|  | 128 | PLD3 | 7.06 | 0.88 | 9.8^-03^ | SKMEL5 | 1.0^-05^ |
| valproic acid | 143 | ATP6V1F | 11.12 | -1.19 | 4.4^-04^ | SKMEL5 | 1.0^-03^ |
|  | 137 | ATP6V1F | 9.88 | 0.71 | 3.5^-02^ | MCF7 | 5.0^-05^ |
|  | 138 | ATP6V1F | 9.87 | 0.58 | 9.3^-02^ | MCF7 | 5.0^-05^ |
|  | 139 | PLD3 | 7.58 | 1.18 | 9.8^-03^ | MCF7 | 1.0^-03^ |
|  | 140 | PLD3 | 7.54 | 0.91 | 3.3^-02^ | MCF7 | 2.0^-03^ |
|  | 143 | PLD3 | 7.03 | 0.70 | 3.3^-02^ | SKMEL5 | 1.0^-03^ |
| verapamil | 275 | PLD3 | 7.28 | 0.91 | 5.5^-02^ | MCF7 | 1.0^-05^ |
| wortmannin | 149 | ATP6V1F | 11.13 | -1.10 | 4.4^-04^ | SKMEL5 | 1.0^-08^ |
|  | 150 | NFE2 | 5.10 | 2.21 | 1.2^-03^ | ssMCF7 | 1.0^-08^ |
| yohimbine | 151 | ETS2 | 6.21 | 1.40 | 6.5^-02^ | MCF7 | 2.30^-05^ |
